# Angiosarcoma patients treated with immune checkpoint inhibitors: a case series of seven patients from a single institution

**DOI:** 10.1186/s40425-019-0689-7

**Published:** 2019-08-08

**Authors:** Vaia Florou, Andrew E. Rosenberg, Eric Wieder, Krishna V. Komanduri, Despina Kolonias, Mohamed Uduman, John C. Castle, Jennifer S. Buell, Jonathan C. Trent, Breelyn A. Wilky

**Affiliations:** 1 Department of Medicine, Division of Oncology, Sylvester Comprehensive Cancer Center at University of Miami Miller School of Medicine, 1475 NW 12th Avenue, Miami, FL 33136 USA; 2 Agenus Inc., 3 Forbes Road, Lexington, MA 02421 USA

**Keywords:** Angiosarcoma, Checkpoint inhibitors, CTLA-4 antibody, Fusions, Tumor mutation burden

## Abstract

**Background:**

Angiosarcoma is an uncommon endothelial malignancy and a highly aggressive soft tissue sarcoma. Due to its infiltrative nature, successful management of localized angiosarcoma is often challenging. Systemic chemotherapy is used in the metastatic setting and occasionally in patients with high-risk localized disease in neoadjuvant or adjuvant settings. However, responses tend to be short-lived and most patients succumb to metastatic disease. Novel therapies are needed for patients with angiosarcomas.

**Methods:**

We performed a retrospective analysis of patients with locally advanced or metastatic angiosarcoma, who were treated with checkpoint inhibitors at our institution. We collected their clinical information and outcome measurements. In one patient with achieved complete response, we analyzed circulating and infiltrating T cells within peripheral blood and tumor tissue.

**Results:**

We have treated seven angiosarcoma (AS) patients with checkpoint inhibitors either in the context of clinical trials or off label [Pembrolizumab + Axitinib (NCT02636725; *n* = 1), AGEN1884, a CTLA-4 inhibitor (NCT02694822; *n* = 2), Pembrolizumab (*n* = 4)]. Five patients had cutaneous angiosarcoma, one primary breast angiosarcoma and one radiation-associated breast angiosarcoma. At 12 weeks, 5/7 patients (71%) had partial response of their lesions either on imaging and/or clinical exam and two (29%) had progressive disease. 6/7 patients are alive to date and, thus far, 3/7 patients (43%) have progressed (median 3.4 months)- one achieved partial response after pembrolizumab was switched to ongoing Nivolumab/Ipilimumab, one died of progressive disease at 31 weeks (primary breast angiosarcoma) and one was placed on pazopanib. One patient had a complete response (CR) following extended treatment with monotherapy AGEN1884. No patient experienced any ≥ grade 2 toxicities.

**Conclusions:**

This case series underscores the value of targeted immunotherapy in treating angiosarcoma. It also identifies genetic heterogeneity of cutaneous angiosarcomas and discusses specific genetic findings that may explain reported benefits from immunotherapy.

## Background

Angiosarcoma is an uncommon and highly aggressive sarcoma in which the neoplastic cells exhibit endothelial differentiation. As many as 60% of angiosarcomas are cutaneous and usually present in the tissues of the scalp, face, and neck, but can arise in any part of the body [1]. Surgical resection is the main therapy for localized disease, but due to the infiltrative growth pattern, resection with sufficient margins is often challenging particularly in head and neck locations and postoperative recurrence and eventual metastases are frequent.

Initial responses to cytotoxic chemotherapy are common, but the duration of response is often limited, and most patients eventually succumb to metastatic disease. Complete responses of angiosarcomas can occasionally occur with chemotherapy, including taxanes and doxorubicin-based regimens [2, 3], but few effective therapies exist for patients who progress on these agents. With a median overall survival of only 30–50 months [4], novel therapies for angiosarcoma are needed.

Immune checkpoint inhibitors (ICIs) are currently in clinical trials that include angiosarcoma patients (NCT02815995). Case reports in the literature have shown remarkable response of visceral and cutaneous angiosarcoma involvement in patients treated with an anti-PD-1 antibody [5, 6]. The Angiosarcoma Project, a patient-led effort to obtain genetic sequencing of angiosarcoma samples, has demonstrated that cutaneous angiosarcomas may possess UV mutation signatures as found in melanoma [7]. Given the high mutation burden in melanoma, and the relatively high response rates to modern immunotherapy, this offers a potential hypothesis to explain early evidence of PD-1 blockade activity in cutaneous angiosarcomas.

Herein, we present a series of patients with chemotherapy-refractory angiosarcomas who were treated with checkpoint inhibitors.

### Case series

We identified seven patients with angiosarcoma treated with ICIs on clinical trials or off-label since 2016 (Table 1). Most patients had cutaneous angiosarcoma (5/7), one had primary breast angiosarcoma and one radiation-associated breast angiosarcoma. Among the patients with cutaneous angiosarcoma (*n* = 5), three had scalp and two facial involvement. The median age was 68 years and 5/7 patients were females. Patients had either metastatic or locally advanced disease with multiple recurrences. All patients had received prior systemic therapies (range 1–6, mean 3), and received 4–14 doses of ICIs (median 5).

**Table 1:** Patient demographics and characteristics. cAS cutaneous angiosarcoma, RAS radiation associated angiosarcoma

Patient	Demographics	Pathology	Disease state	Prior therapies	Immunotherapy	Number of ICI doses	Response at 12 weeks	Duration of response to ICI	Best Overall Response	Post ICI therapies
1	32-year-old female	Primary breast AS	MetastaticSoft tissue, Bones	Gemcitabine/ Docetaxel	Axitinib + Pembrolizumab (NCT02636725)	4	Progression of disease		Progression of disease	Paclitaxel Doxorubicin/ Olaratumab
2	71-year-old female	Breast RAS	Metastatic Mediastinal lymph nodes, Lung	Doxorubicin/ Olaratumab, Gemcitabine/ Docetaxel, Pazopanib	Pembrolizumab	5	Partial response	Ongoing	Partial response	N/A
3	62-year-old female	cAS	Locally advanced- face	Doxorubicin, Gemcitabine/ Docetaxel, Pazopanib, Ifosfamide, Notch Inhibitor (NCT01695005), Temozolamide/ Bevacizumab	Anti-CTLA-4 (NCT02694822)	14	Partial response	Ongoing	Complete response	None
4	68-year-old female	cAS	Metastatic Lymph nodes, Bones	IL-2/ Cyclophosphamide/ Methotrexate, Paclitaxel, Bevacizumab	Pembrolizumab	6	Partial response^b^	14 weeks	Partial response^b^	N/A
					Ipilimumab/Nivolumab	8	Partial response^b^	Ongoing	Partial response^b^	N/A
5	89-year-old female	cAS	Multifocal- scalp	Gemcitabine, Paclitaxel, Pazopanib	Pembrolizumab	5	Partial response^a^	Ongoing	Partial response	None
6	76-year-old male	cAS	Multifocal- scalp	Pazopanib/TRC105 (NCT02979899), Doxorubicin/ Cyclophosphamide/ Olaratumab, Gemcitabine/ Docetaxel	Pembrolizumab	5	Partial response^a^	Ongoing	Partial response	N/A
7	65-year-old male	cAS	Multifocal- nose	Doxorubicin/ Ifosfamide, Doxorubicin/ Cyclophosphamide, Gemcitabine/ Docetaxel	Anti-CTLA-4 (NCT02694822)	7	Progression of disease		Progression of disease	Pazopanib

^a^Response assessed by clinical exam only ^b^Response assessed by clinical exam and imaging

Response to therapy was evaluated every 2 to 3 months by radiographic imaging as well as by physical examination. Patients with measurable disease by RECIST 1.1 criteria were assessed for response by comparing baseline and on-treatment imaging, with partial response (PR) requiring greater than 30% decrease in the sum of greatest diameter of target tumor lesions. Patients with cutaneous involvement not assessible by radiographic imaging were monitored as non-target lesions by RECIST 1.1 guidelines.

Five out of seven patients achieved partial or complete response as best overall response. Patient 3 met RECIST 1.1 criteria for complete response (CR) and patient 2 for PR. Patients 5 and 6 had PR based on evaluation of cutaneous, non-measurable lesions. Patient 4 achieved PR based on RECIST 1.1 criteria as well as based on cutaneous lesion measurements (Table 1).

**Fig. 1:**
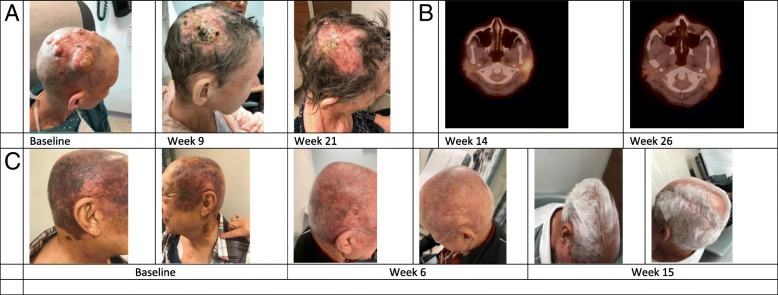
**a.** Clinical photographs of patient 4. **b.** PET imaging showing the site of progression of patient 4 before and after switching therapy to Nivolumab/Pembrolizumab. **c.** Clinical photographs of patient 6

Three patients (patient 2, 4 and 6) are presently on ICI with continuing response (Fig. 1c, Table 1). Patient 5 (cutaneous angiosarcoma) discontinued Pembrolizumab due to personal preference. Patient 3 (cutaneous angiosarcoma) received 14 doses of AGEN1884, an anti-CTLA-4 antibody at a low dose of 0.1 mg/kg, and achieved CR by imaging, physical exam and biopsy. Patient remains in CR more than 1 year off therapy.

By 12 weeks after initiating ICIs, 2/7 (28%) patients met radiographic criteria for progressive disease; patient 1 with primary breast angiosarcoma and patient 7 with cutaneous multifocal angiosarcoma. Patient 1 was switched to another therapy shortly after progressing to Pembrolizumab but eventually succumbed to her disease 31 weeks after initiation of ICI. For patient 7, therapy was continued for two additional doses past radiographic progression per protocol, and was changed to Pazopanib when confirmed progressive disease. One patient with metastatic cutaneous angiosarcoma (patient 4), developed a mixed response at 14 weeks on pembrolizumab, with some lesions improving whereas others were worsening (Fig. 1a), but achieved partial response after changing therapy to Ipilimumab/Nivolumab (Fig. 1b). The median duration of response to ICI for the three patients who progressed was 3.4 months and not reached for the rest. No patient developed any grade 3 or 4 immune related adverse effects (irAE).

### Correlative studies

**Fig. 2:**
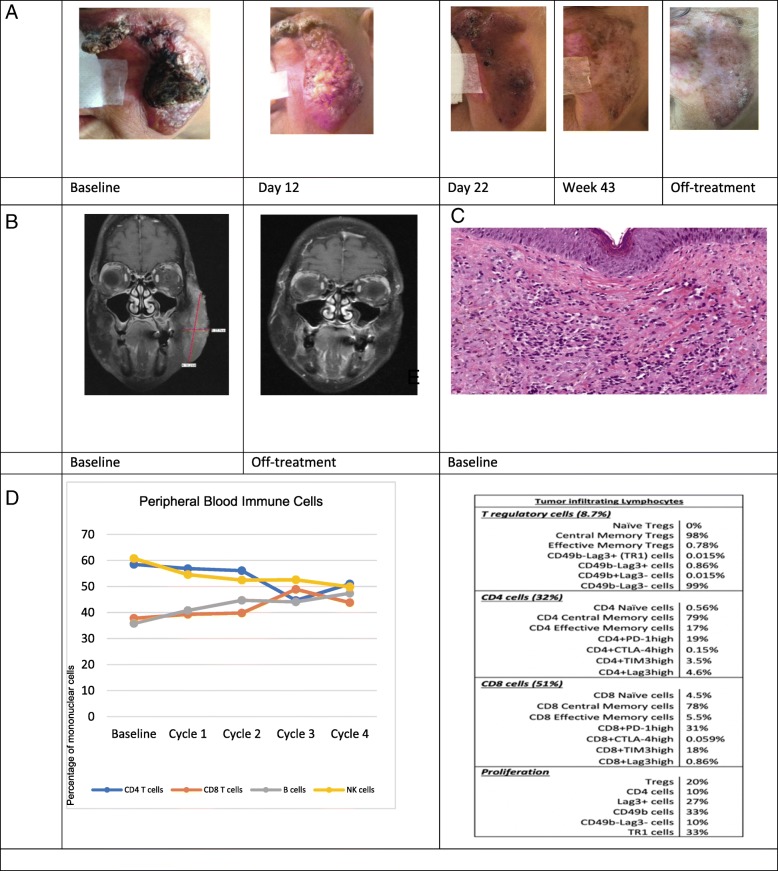
(Patient 3) **a**. Clinical photographs of cutaneous angiosarcoma lesion before and after treatment with AGEN1884, a monoclonal antibody to immune checkpoint CTLA-4. **b**. Magnetic resonance imaging before and after treatment with AGEN1884. **c**. Immunohistologic appearance of angiosarcoma showing malignant cells that line poorly-formed vascular lumens and infiltrate the dermis. **d**. Relative proportions of circulating immune cells within the peripheral blood at baseline and with subsequent treatments with AGEN1884. **e**. Immune phenotyping by multiparameter flow cytometry of tumor-infiltrating lymphocytes isolated from angiosarcoma tissue biopsy 12 days after the first dose of AGEN1884

Based on the remarkable activity of ICIs in angiosarcoma patients, we performed exploratory analysis of immune and genetic features of patient 3 who achieved CR on a Phase I clinical trial of anti-CTLA4 antibody AGEN1884 (NCT02694822) (Fig. 2). AGEN1884 is a fully human monoclonal immunoglobulin G1 κ subclass (IgG1κ) antibody that specifically recognizes CTLA-4 and mediates strong inhibition of CTLA-4:CD80/CD86 axis [8]. While of the same IgG1 class as ipilimumab, preclinical data suggests this molecule may have enhanced activity against T regulatory cells. We obtained a core needle tumor biopsy 12 days after the first dose of AGEN1884, and isolated tumor infiltrating lymphocytes (TILs) by flow cytometry, with attention to CD8^+^, CD4^+^, and T regulatory cells (Fig. 2e). CD4^+^ T cells consisted of central memory T cells (79%) and effector memory T cells (17%), whereas CD8^+^ T cells consisted of central memory T cells (78%) and less of effector memory T cells (5.5%). Both CD4^+^ and CD8^+^ T cells expressed PD-L1 as 17 and 31%, respectively. Tregs had a predominantly central memory phenotype, with an almost absent population of CD49b^+^Lag3^+^ (TR1) cells which typically represent an exhausted T cell phenotype. Expression of other checkpoint receptors is noted in Fig. 2e.

Additionally, mononuclear cells from peripheral blood of patient 3 were isolated on the first day of the first four cycles (Fig. 2d). Two populations decreased from baseline: the natural killer cells (NK) and CD4^+^ T cells. However, both CD4^+^ T cells and NK cells were above 50% of the peripheral blood immune cells at baseline. Peripheral B cells and CD8^+^ T cells increased above baseline with treatment, from 35.8 to 47.5% and from 37.8 to 43.8%, respectively.

Further, we measured numerous cytokines on peripheral blood of Patient 3 at different time points during the first two cycles. Measurable cytokines were IL-6, IL-8, TNF and IFN-γ. Interestingly, IFN-γ and IL-6 decreased relative to baseline before the second cycle and remained as such 7 days after but no particular response pattern was observed in the other measurable cytokines.

Lastly, next-generation whole exome (WES) and RNA sequencing (RNA-Seq) were performed on archival tumor tissue from this patient’s original surgery (rhinectomy), and WES on blood-extracted DNA. Comparison of tumor and normal DNA identified 287 tumor mutations, with only 6 missense mutations. Missense mutations occurred in the genes *NBPF10*, *NBPF15*, *ZNF678*, *VPS8*, *PCLO* and *ABCB1*. The function as well as the exact clinical relevance of these genes are not known in sarcomas. Mutations in the *PLCO* gene have been found in poorly differentiated hepatocellular carcinoma [9] as well as in hematological malignancies [10], however its role in the pathogenesis of either is not known. The *NBPF* genes encode proteins whose function are still not fully understood, but have been shown to be highly expressed in sarcoma with unknown clinical or prognostic relevance [11, 12]. Interestingly, the *ABCB1* gene has been implicated in the export of taxanes and other cytotoxic agents, and gene polymorphisms have been shown to have both predictive value in ovarian cancer [13]. The overall tumor mutation burden (TMB) was low at only 0.09 mutations/mb. Multiple putative fusion transcripts were identified, including 31 fusions predicted to generate novel protein sequences. Additionally, 20 of 246 genes associated with cancer testis antigens [14] were expressed at over 1 FPKM (fragments per Kilobase per million) in the tumor RNA-Seq data.

Sufficient archived tumor tissue was unavailable for the other patients treated in the study for correlative analysis. However, patients 4 and 5, both of whom achieved partial response, had previously undergone tumor comprehensive genomic profiling (CGP) performed by FoundationOne™. Both patients had intermediate tumor mutation burden based on pre-established histology nonspecific cutoffs per FoundationOne™. Remaining genomic findings from these two patients are listed in Table 2.

**Table 2:** Comprehensive genomic profiling by FoundationOne™

Patient	TMB	Genomic Findings
4	15 muts/mb	CDKN2A/B loss CHEK2 R117G DNMT3A R771 FANCD2 E118 MLL3 splice site 2533-1G > A TP53 L145R, splice site 673-1G > A
5	12 muts/mb	CRKL amplification DNMT3A R635W – subclonal MAPK1 amplification SF3B1 K700E – subclonal ZRSR2 splice site 121 + 2 T > G

## Discussion

Checkpoint inhibition may be effective in a subset of patients with soft tissue sarcomas. In the SARC028 Phase 2 study of the anti-PD1 antibody pembrolizumab, objective response rates (ORR) of 18% (soft tissue) and 5% (bone) were observed in 84 patients with advanced or metastatic soft tissue and bone sarcoma [15]. While anti-CTLA-4 inhibitor ipilimumab monotherapy in patients with synovial sarcoma was disappointing with no responses observed in six patients, leading to study closure [16], ipilimumab in combination with nivolumab (anti PD-1) produced ORR of 16% compared to 5% with nivolumab monotherapy [17]. Three angiosarcoma patients were included in the latter study, all in the combination arm with one confirmed response. Numerous studies combining CTLA-4 inhibition with immunotherapy, tyrosine kinase inhibitors, or chemotherapy are ongoing for sarcoma patients (ie Trabectedin, Ipilimumab and Nivolumab (NCT03138161), Ipilimumab and Imatinib (NCT01738139).

To our knowledge, we report the first complete response in angiosarcoma to CTLA-4 monotherapy. Since this patient was heavily pretreated, including prior radiation, we hypothesized that an explanation for her remarkable response might be found in the tumor mutational profiling. The genetic heterogeneity of angiosarcomas has been the subject of various studies, focused primarily on radiation-associated angiosarcomas. In a pooled radiation-associated and sporadic angiosarcoma analysis, a subset of patients shared a mutational signature of UV light (preponderance of C → T substitutions) similar to UV-associated skin carcinoma of the scalp [18]. Findings from the Angiosarcoma Project also suggest that some cutaneous angiosarcomas have analogous genetic backgrounds to UV light-related skin cancer. UV mutation signature and overall mutation burden in melanoma patients confer clinical benefit to CTLA-4 inhibition [19], but not to adoptive cellular therapies [20].

Mutational burden has predicted response to checkpoint inhibitors in other cancer types, especially in cancers with microsatellite instability (MSI). These tumors may exhibit thousands of mutations, and respond exceedingly well to checkpoint inhibitors, leading to multiple FDA indications for cancers with microsatellite instability [21]. Outside of MSI, mutational burden has also correlated with response to checkpoint inhibitors in various solid cancers [22]. Recently though, it has been appreciated that tumor mutational burden might not be sufficient to predict response to immunotherapy in all patients. In contrast, the neoantigen signature and its immunogenicity appears more important in predicting the response to checkpoint inhibition and adoptive T cell therapy in melanoma patients [20]. While insufficient alone, the tumor mutational load can certainly increase the probability of neoantigen signature and response to checkpoint inhibition.

In the exceptional responder in our series (patient 3), the tumor mutational burden was surprisingly low, thus one may not have anticipated the remarkable response to extremely low dosing of anti-CTLA-4 antibody. The tumor, however, did express many novel protein fusions and cancer-testis antigens. Our patient’s remarkable clinical response suggests tumor immunogenicity, which was not reflected merely by the number of mutations per megabase. Another emerging biomarker of response to immunotherapy is the mutation subtypes. A pan-cancer analysis showed that increased numbers of indel frameshift mutations in renal cell carcinoma and melanoma are associated with higher cytotoxic T cell infiltration, higher neoantigen formation, and better immunotherapy responses [23]. Similarly, gene fusions can generate peptides which may eventually serve as neoantigens and elicit immunogenic responses. Another pan-cancer analysis from TCGA (The Cancer Genome Atlas) database showed 1.5 predicted peptides per fusion across different cancer types, with frameshift fusions generating more immunogenic epitopes than in-frame fusions [24]. These findings are particularly important, because some patients with driver fusions may be excluded from checkpoint inhibitor trials due to their low tumor mutation burden.

The immune tumor microenvironment of patient 3 consisted of mainly central memory CD4^+^ and CD8^+^ T cells, and Tregs. While we do not have baseline TIL profiling prior to treatment, these findings can potentially suggest the importance of memory T cell subtypes in mediating robust effector functions upon re-exposure to antigens and maintaining this patient’s durable response. We can only postulate that the prior chemotherapy and radiation might have generated more neoantigens potentially driven by fusions, promoting expansion of these memory T cells, eliciting cytotoxic reactions with checkpoint inhibition and perhaps that AGEN1884 may have augmented this antigen-specific immune response [8]. The effector/memory Tregs play a key role in the loss of tumor immunity, even in the presence of cytotoxic CD8^+^ T cells, and they have greater effector functions and higher CTLA-4 expression in preclinical studies [25].

However, whether the high expression of PD-L1 (31%) and TIM3 (18%) on CD8^+^ T cells as well as the PD-L1 expression on CD4^+^ T cells (19%) in patient’s 3 biopsy, relatively to the very low CTLA-4 expression in both T cell populations could be attributed to therapy effect is unclear due to lack of pre-therapy available tissue. Nevertheless, PD-L1 positivity neither in tumor cells nor in TILs, was required in ICI responders in sarcoma trials [15, 26].

Another interesting finding from the correlative studies of patient 3 was the high percentage of circulating NK cells and CD4^+^ T cells at baseline. Data on CTLA-4 expression on NK cells are limited. Studies in melanoma and lung cancer mouse models suggest that NK responses could be mediated through the CD28/CTLA-4:B7–1/B7–2 system, by direct inhibition of NK IFN-γ production [27]. Thus, CTLA-4 inhibition could indirectly enhance NK effector functions. Peripheral B and CD8^+^ T cells increased above baseline, suggesting augmentation of cytotoxic T and B cells as expected by inhibition of CTLA-4.

Lastly, we did not observe any pattern of alterations in serum cytokines including IFN-γ, IL-6 and TNF, perhaps due to limited time points. High pre-treatment IL-8 has negative predictive value, as this pro-inflammatory cytokine might play a role in the immune escape. Decreases from baseline in melanoma patients, have been associated with improved responses to anti- CTLA-4 immunotherapy [28]. In our patient, IL-8 levels overall were fluctuating with downward trends after each dose.

While intriguing, our results are limited by the retrospective nature of the study, and the heterogeneity within included angiosarcoma subtypes, treatment regimens, and response assessment measures. Additionally, our ability to perform additional correlative studies was limited by the available tissue reserves for studied patients. Thus, our findings are meant to be viewed as hypothesis-generating, and require further investigation in a prospective clinical trial.

## Conclusion

In this series, we report intriguing evidence of efficacy of ICIs in patients with angiosarcoma, including the first report of a complete response in a patient with cutaneous angiosarcoma treated with CTLA-4 inhibition as monotherapy. While angiosarcoma is a genetically heterogenous disease, increasing evidence suggests that cutaneous angiosarcomas share genetic similarities with UV light exposed cancers and may benefit from checkpoint inhibition. In our patient cohort and in particular in patient 3, it is unclear to what degree prior therapies altered the tumor microenvironment to subsequently sensitize them to checkpoint inhibition. Further studies are critical to better characterize the immune microenvironment of angiosarcomas, especially the effects with traditional therapies, which will shed light on mechanisms of response and reveal new targets for repolarization of the immunosuppressive tumor environment towards an anti-tumor phenotype. Given the paucity of treatment options in these devastating sarcomas, our patients’ responses give hope that checkpoint inhibitors could eventually replace or augment traditional treatment strategies. Ultimately, the promising activity of ICIs against angiosarcoma warrant a randomized prospective study to confirm efficacy in chemorefractory patients.

## Materials and methods of the correlative studies (patient 3)

### Characterization of TILs

biopsy specimens were processed in real time and a single cell suspension was made by digesting tissue with dispase/collagenase (Roche Liberase DH) in media with addition of DNAse at 37 degrees, and single cells isolated using a Ficoll gradient. Cells were stained with antibodies, followed by FACS analysis for profiling. The antibodies used for FACS: CD3 Alexa, CD4 APC cy7, CD25 BV650, CD27 PE-CF594, CD45RA BV421, CTLA4(CD152) BV786 and PD-1 BB515 were purchased from BD Biosciences; LIVE/Aqua BV510 from Invitrogen; CD8 BV570 from Biolegend; CD127 PE from Beckman Coulter; Lag3 PE-Cy7 and TIM-3 APC from eBioscience.

### Analysis of circulating peripheral blood mononuclear cells

samples were processed by Ficoll gradient to isolate serum, peripheral mononuclear cells (PBMC), and plasma. PBMCs were stained with antibodies followed by FACS analysis.

### Measurement of cytokines

Luminex Multiple assays were used.

### Genetic profiling of tumor

WES of tumor and matched patient PBMCs were conducted by Personalis Inc. using the Personalis ACE Exome™ Assay (ACE v3). Personalis Cancer DNA Pipeline was used to identify tumor somatic variants, short insertions and deletions. Similarly, RNA-Seq was performed using Personalis ACE Transcriptome™ Assay (ACE v3), and the Personalis Cancer RNA Pipeline was used for gene expression analysis. All sequencing reads were aligned to hs37d5 reference genome build. The analysis pipeline performs alignment, duplicate removal, and base quality score recalibration using best practice guidelines recommended by the Broad Institute.

### Tumor mutation burden

Calculated as the number of non-synonymous somatic mutations per DNA megabase, as derived from the WES-based mutation discovery.

### Gene fusion detection

After filtering the RNA-Seq reads for quality and removal of bacterial and viral sequences, bioinformatic tools were used to identify gene fusions [29–31].

*Performed by Agenus.

## Abbreviations

AS: angiosarcoma
CGP: comprehensive genomic profiling
CR: complete response
FPKM: fragments per Kilobase per million
ICI: Immune checkpoint inhibitors
irAE: immune related adverse effects
MSI: microsatellite instability
PBMC: peripheral mononuclear cells
RNA-Seq: RNA sequencing
T regs: T regulatory cells
TCGA: The Cancer Genome Atlas
TILs: tumor infiltrating lymphocytes
TMB: tumor mutation burden
WES: whole exome sequencing
