# Correction to: Angiosarcoma patients treated with immune checkpoint inhibitors: a case series of seven patients from a single institution

**DOI:** 10.1186/s40425-019-0792-9

**Published:** 2019-11-06

**Authors:** Vaia Florou, Andrew E. Rosenberg, Eric Wieder, Krishna V. Komanduri, Despina Kolonias, Mohamed Uduman, John C. Castle, Jennifer S. Buell, Jonathan C. Trent, Breelyn A. Wilky

**Affiliations:** 1 Department of Medicine, Division of Oncology, Sylvester Comprehensive Cancer Center at University of Miami Miller School of Medicine, 1475 NW 12th Avenue, Miami, FL 33136 USA; 2 Agenus Inc., 3 Forbes Road, Lexington, MA 02421 USA

**Correction to: J ImmunoTher Cancer**

**https://doi.org/10.1186/s40425-019-0689-7**

Following publication of the original article [1], the authors have reported that the following sentence “While of the same IgG1 class as ipilimumab, preclinical data suggests this molecule may have enhanced activity against T regulatory cells.”

should be replaced with

“Ipilimumab and AGEN1884 are IgG1 class antibodies. AGEN1884 data show CTLA-4 inhibition and activity against T regulatory cells (reference 8).”

## References

[1] Florou (2019). Angiosarcoma patients treated with immune checkpoint inhibitors: a case series of seven patients from a single institution. J ImmunoTher Cancer.

